# Shannon Capacity and Related Graph Invariants for Lexicographic Products

**DOI:** 10.3390/e28090994

**Published:** 2026-09-05

**Authors:** Igal Sason

**Affiliations:** 1 Department of Electrical and Computer Engineering, Technion–Israel Institute of Technology, Haifa 3200003, Israel; sason@ee.technion.ac.il; Tel.: +972-48294699; 2 Department of Mathematics, Technion–Israel Institute of Technology, Haifa 3200003, Israel

**Keywords:** lexicographic product, strong product, Shannon capacity of graphs, zero-error information theory, graph invariants, Lovász theta function, fractional Haemers number, 05C35, 05C50, 05C69, 05C76, 94A15

## Abstract

This paper studies the Shannon capacity of lexicographic products of finite simple graphs, together with the Lovász theta function and the fractional Haemers number. The Shannon capacity is proved to be supermultiplicative under lexicographic products in either order, and these products are compared with the strong product. We explicitly construct three countably infinite families of lexicographic powers based on the Schläfli graph, the McLaughlin graph, and its second subconstituent; in each family, pairing each member with its complement yields strict supermultiplicativity and arbitrarily large multiplicative gaps. Bounds and exact-capacity criteria for lexicographic products are derived, and the resulting upper bounds are shown to be incomparable. The capacities of lexicographic products involving Kneser graphs, their complements, and *q*-analogues of Kneser graphs are determined. It is also shown that a lexicographic product with a complete outer factor preserves the Shannon capacity of an arbitrary inner factor. The capacities of iterated lexicographic powers are determined, including those of self-complementary graphs that are vertex-transitive or strongly regular. Elementary, self-contained proofs are also given for three known results: the multiplicativity of the Lovász theta function and the fractional Haemers number under lexicographic products, and the equality of the fractional and ordinary Lovász theta functions. Finally, an open problem concerning the Shannon capacities of lexicographic and strong products is posed.

## 1. Introduction

The lexicographic product is a fundamental graph operation that arises naturally by replacing each vertex of one graph with a copy of another. For finite simple graphs *G* and *H*, their *lexicographic product*, denoted by G∘H or G[H], has the vertex set V(G)×V(H), where two distinct vertices (g,h) and $\left(g^{\prime },h^{\prime }\right)$ are adjacent if and only if

$$g{∼}_{G}g^{\prime }\;\vee \;\left(g=g^{\prime }\;\wedge \;h{∼}_{H}h^{\prime }\right).$$

Here, the relations ${∼}_{G}$ and ${∼}_{H}$ denote adjacency in *G* and *H*, respectively. Equivalently, each vertex g∈V(G) is replaced by a copy of *H*, and whenever $g{∼}_{G}g^{\prime }$, every vertex in the copy corresponding to *g* is joined to every vertex in the copy corresponding to $g^{\prime }$.

The lexicographic product provides a natural setting for studying the behavior of several important graph invariants, including the Lovász theta function, the fractional Haemers number, and the Shannon capacity. It also admits a natural interpretation in zero-error information theory. To formulate this interpretation, we first recall the graph-theoretic description of zero-error communication.

Given a discrete memoryless channel with input alphabet X, its *confusability graph G* has vertex set X, with two distinct input symbols adjacent if they can produce a common channel output and therefore cannot be distinguished with zero-error probability [1]. Thus, a collection of pairwise nonconfusable symbols forms an independent set in *G*. Consequently, the maximum number of messages that can be transmitted without error in a single use of the channel is α(G), the *independence number* of *G*, defined as the maximum cardinality of an independent set in *G*.

For *n* channel uses, the resulting confusability graph is the *n-fold strong power* $G^{⊠n}$, whose vertex set is $V{\left(G\right)}^{n}$, with two distinct vertices adjacent if, in each coordinate, their entries are either identical or adjacent in *G*. Consequently, the maximum number of messages that can be transmitted without error in *n* channel uses is $\alpha (G^{⊠n})$. The *Shannon capacity* of *G* is defined by

(1) $$\begin{array}{cc} \Theta (G) & ≔\underset{n\geq 1}{\sup}\sqrt[{n}]{\alpha \left(G^{⊠n}\right)}\\ \; & =\lim_{n\to \infty }\sqrt[{n}]{\alpha \left(G^{⊠n}\right)}, \end{array}$$

and the limit exists and equals the supremum by Fekete’s lemma, since

(2) $$\begin{array}{c} \alpha \left(G^{⊠(m+n)}\right)\geq \alpha \left(G^{⊠m}\right)\;\alpha \left(G^{⊠n}\right),\;n,m\geq 1. \end{array}$$

Thus, Θ(G) is the asymptotic per-channel-use growth factor of the maximum number of messages that can be transmitted with zero error probability, whereas logΘ(G) is the corresponding asymptotic exponential growth rate [1].

Despite the simplicity of its definition, Θ(G) is notoriously difficult to determine. Its exact value remains unknown even for some small simple graphs, such as the cycle of length 7, and the independence numbers of successive strong powers can exhibit remarkably complicated behavior. Alon and Lubetzky [2] showed, in particular, that the Shannon capacity cannot in general be approximated within a subpolynomial factor in the number of vertices using any fixed, but arbitrarily long, initial segment of the sequence

$${\left\{\alpha (G^{⊠n})\right\}}_{n\geq 1}\;.$$

The subject and its connections with graph powers, extremal combinatorics, coding theory, and spectral methods are surveyed from several perspectives in [3,4,5].

A remarkable development was the introduction by Lovász of the theta function ϑ(G), which is computable to arbitrary prescribed accuracy in polynomial time by semidefinite programming and satisfies the sandwich inequalities

(3) $$\begin{array}{c} \alpha \left(G\right)\leq \Theta \left(G\right)\leq \vartheta \left(G\right)\leq \chi \left(\bar{G}\right). \end{array}$$

Thus, ϑ(G) provides an upper bound on the Shannon capacity of *G* and a lower bound on the chromatic number of its complement $\bar{G}$ [6]. In particular, by combining the upper bound $\vartheta \left(C_{5}\right)=\sqrt{5}$ with the standard construction showing that $\alpha (C_{5}^{⊠2})=5$, Lovász obtained the celebrated identity

$$\Theta \left(C_{5}\right)=\sqrt{5}.$$

The Lovász theta function also lies at the heart of the sandwich theorem and the interplay among orthonormal representations and semidefinite optimization [6,7,8], graph parameters [7,9,10], and coding-theoretic bounds [11,12,13,14]. Another important upper bound on the Shannon capacity is Haemers’ linear-algebraic minimum-rank bound [15], along with its fractional variant [16]. Hu, Tamo, and Shayevitz subsequently developed a linear-programming bound that can improve upon both the Haemers minimum-rank bound and the Lovász theta bound [17]; see also [5,10,18] for discussions of theta-type, spectral, and related graph invariants.

We next describe the information-theoretic interpretation of the lexicographic product. Let *G* and *H* be finite confusability graphs, and suppose that the channel input alphabet is V(G)×V(H). Here, g∈V(G) serves as a *class label*, whereas h∈V(H) is a *within-class symbol*. For each g∈V(G), let

$$H_{g}≔\left\{(g,h):h\in V(H)\right\}.$$

Assume that the confusability graph induced on each class $H_{g}$ is a copy of *H*, and that the confusability relations between distinct classes depend only on their class labels. More precisely, for $g\neq g^{\prime }$, every input in $H_{g}$ is confusable with every input in $H_{g^{\prime }}$ if $g{∼}_{G}g^{\prime }$, whereas no input in $H_{g}$ is confusable with any input in $H_{g^{\prime }}$ if $g{≁}_{G}g^{\prime }$. It follows that

(4) $$\begin{array}{c} \left(g,h\right)∼\left(g^{\prime },h^{\prime }\right)\;⟺\;\left(g{∼}_{G}g^{\prime }\right)\;\vee \;\left(g=g^{\prime }\;\wedge \;h{∼}_{H}h^{\prime }\right). \end{array}$$

Thus, the confusability graph of the channel is exactly G∘H.

The uniformity of the confusability relations between distinct classes is an explicit assumption of this model, rather than a consequence of representing the channel inputs as ordered pairs. If within-class symbols also affected confusability between distinct classes, the resulting graph would not, in general, be a lexicographic product. Accordingly, Θ(G∘H) is the zero-error capacity of this class-structured channel.

**Example** **1.** *Let G=KG(n,k) and H=KG(m,ℓ) be Kneser graphs, where n≥2k and m≥2ℓ; for background on these graphs, see Section 2.5. The channel inputs are pairs:*

(5) (A,B)∈[n]k×[m]ℓ.

*Two distinct inputs (A,B) and $\left(A^{\prime },B^{\prime }\right)$ are confusable if and only if*

(6) $$\begin{array}{c} \left(A\cap A^{\prime }=⌀\right)\;\vee \;\left(A=A^{\prime }\;\wedge \;B\cap B^{\prime }=⌀\right). \end{array}$$

*Hence, the confusability graph is the lexicographic product*

KG(n,k)∘KG(m,ℓ),

*whose Shannon capacity is determined in Theorem 8.*

Lexicographic products also arise naturally in index coding and network coding, where they provide a structured means of constructing larger coding problems from smaller ones. Blasiak, Kleinberg, and Lubetzky represented index-coding instances by directed hypergraphs and defined a lexicographic product for such instances. By taking repeated lexicographic products, they constructed families exhibiting polynomially growing gaps between various combinatorial bounds and achievable coding rates, including a polynomial separation between linear and nonlinear network-coding rates [19]. Arbabjolfaei and Kim subsequently proved that the broadcast rate is multiplicative under the lexicographic product of side-information graphs [20]. They later introduced a generalized lexicographic product for directed side-information graphs and characterized the capacity region of the composite index-coding problem in terms of the capacity regions of its constituent subproblems [21]; see also the monograph [22] for a comprehensive treatment of index coding. Whereas these applications concern the construction and decomposition of coding problems, the present work studies the Shannon capacity of undirected lexicographic products, with the factor graphs interpreted as confusability graphs.

The purpose of this paper is to study the Shannon capacity and related graph invariants for lexicographic products. Our results fall into two categories. First, we present alternative, elementary, and self-contained proofs of several known results concerning the Lovász theta function and the fractional Haemers number. Second, we derive new bounds and establish strict supermultiplicativity results for lexicographic products, determine the exact Shannon capacity for several families of such products, and pose an open problem concerning the relationship between the Shannon capacities of lexicographic and strong products.

We begin by recording structural properties of the lexicographic product that will be used in the subsequent analysis, including the multiplicativity of the independence and clique numbers. We then provide alternative, elementary, and self-contained proofs of the multiplicativity of the Lovász theta function and the fractional Haemers number under lexicographic products, and of the equality between the fractional and ordinary Lovász theta functions. Although these three results are known, the proofs presented here are included for their simplicity and because they provide a self-contained foundation for the subsequent study of Shannon capacity.

The principal new results concern the Shannon capacity. We first prove that it is supermultiplicative under the lexicographic product:

(7) $$\begin{array}{c} \Theta (G\circ H)\geq \Theta (G)\;\Theta (H). \end{array}$$

Because the lexicographic product is generally noncommutative, the graphs G∘H and H∘G need not be isomorphic and must be considered separately. After the natural identification of their vertex sets, G⊠H is a spanning subgraph of both G∘H and H∘G. We consequently obtain

(8) $$\begin{array}{c} \max\left\{\Theta (G\circ H),\;\Theta (H\circ G)\right\}\leq \Theta \left(G⊠H\right). \end{array}$$

A central result of this paper shows that the supermultiplicativity inequality for Θ in (7) can be strict. We explicitly construct three countably infinite families of lexicographic powers based on the Schläfli graph, the McLaughlin graph, and its second subconstituent. Pairing each member of these families with its complement yields strict supermultiplicativity of Shannon capacity under the lexicographic product, and arbitrarily large multiplicative gaps are obtained within each family; see Propositions 3 and 4, as well as Theorem 7. These results show, in particular, that the behavior of the Shannon capacity for lexicographic products differs fundamentally from that of the Lovász theta function and the fractional Haemers number, both of which are multiplicative graph invariants.

The comparison with the strong product, together with the multiplicativity of the Lovász theta function and the fractional Haemers number under strong products, yields upper and lower bounds on the capacities of both ordered lexicographic products, as well as sufficient conditions for determining these capacities exactly. In particular, if Shannon capacity is multiplicative under the strong product of *G* and *H*, then it is also multiplicative under both G∘H and H∘G. Exact formulas also follow when the Shannon capacities of the individual factors equal their respective Lovász theta numbers.

The Lovász theta function [6] and the fractional Haemers number [16] provide two potentially different upper bounds on the Shannon capacity of a lexicographic product. We show that these bounds are incomparable: neither dominates the other in general, and either may be sharper, depending on the graphs under consideration. We apply the resulting bounds and exactness criteria to lexicographic products involving Kneser graphs, their complements, and *q*-analogues of Kneser graphs.

We also prove that a complete outer factor preserves the Shannon capacity of the inner factor; equivalently, the join of any finite number of identical copies of a graph has the same Shannon capacity as the graph itself. Finally, we determine the capacities of several families of iterated lexicographic powers, including powers of self-complementary graphs that are vertex-transitive or strongly regular.

Recent developments further illustrate the range of parameters, methods, and constructions in zero-error information theory. Examples include the ρ-capacity motivated by zero-error broadcasting [23], spectral and rank-type bounds for distance powers of graphs [24], Shannon capacity and the Lovász theta number under the Mycielski construction [25], quantum-mechanical and finite-automata methods for bounding graph capacity [26], and zero-error capacities of channels with memory [27].

Lexicographic products occur in a variety of graph-theoretic constructions and provide a natural framework for studying the behavior of graph invariants under substitution and composition. More broadly, graph sums and products have been studied, among others, in [5,28], while the categorical product and related multiplicativity phenomena have been studied in [29]. Asymptotic graph parameters provide a systematic language for analyzing graph operations and their associated asymptotic rates [30,31].

The remainder of the paper is organized as follows. Section 2 reviews the necessary background on lexicographic and strong graph products, the Lovász theta function, the fractional Haemers number, the Shannon capacity, and relevant properties of Kneser, generalized *q*-Kneser, and Paley graphs. Section 3 establishes the multiplicativity of the Lovász theta function under lexicographic products and gives an alternative, elementary, and self-contained proof that the fractional Lovász theta function coincides with the ordinary one. Section 4 gives an alternative, elementary, and self-contained proof of the corresponding multiplicativity result for the fractional Haemers number. Section 5 contains the new results on Shannon capacity, including the general bounds, strict-supermultiplicativity constructions, exactness criteria, and applications to several families of lexicographic graph products and iterated lexicographic powers. Finally, Section 6 formulates an open problem concerning the Shannon capacity under lexicographic and strong products.

## 2. Preliminaries

### 2.1. Lexicographic and Strong Products of Graphs

This subsection recalls the lexicographic and strong products of graphs and compares some of their basic structural properties. We first review their definitions and the relation between their edge sets, and then examine the behavior of the independence and clique numbers under these two products. These properties will be used throughout the paper.

**Definition** **1** (Lexicographic product)**.** *Let G and H be finite simple graphs. The lexicographic product of G and H, denoted by G[H] or G∘H, is the graph with vertex set*

(9) $$\begin{array}{c} V(G\circ H)=V(G)\times V(H), \end{array}$$

*in which two distinct vertices (g,h) and $\left(g^{\prime },h^{\prime }\right)$ are adjacent if and only if*

(10) $$\begin{array}{c} \left(g{∼}_{G}g^{\prime }\right)\;\vee \;\left(g=g^{\prime }\;\wedge \;h{∼}_{H}h^{\prime }\right). \end{array}$$

*For a graph G and an integer n≥1, the n-fold lexicographic power of G, denoted by $G^{\circ n}$, is the lexicographic product of n copies of G.*

For completeness, we recall several elementary structural properties of the lexicographic product. It is associative (see Proposition 5.11 of [32]),

(11) $$\begin{array}{c} (F\circ G)\circ H≅F\circ (G\circ H), \end{array}$$

but it is not commutative in general; that is, G∘H and H∘G need not be isomorphic (see Section 10.3 of [32]). It is right-distributive over disjoint unions,

(12) $$\begin{array}{c} (F+G)\circ H≅(F\circ H)+(G\circ H), \end{array}$$

where + denotes disjoint union, but there is no corresponding left-distributive law. For example (see p. 57 of [32]),

$$K_{2}\circ \left(K_{1}+K_{1}\right)=C_{4},\;\left(K_{2}\circ K_{1}\right)+\left(K_{2}\circ K_{1}\right)=K_{2}+K_{2},$$

where $C_{n}$ and $K_{n}$ denote, respectively, the cycle graph on n≥3 vertices and the complete graph on n≥1 vertices.

It is easily verified that complementation commutes with the lexicographic product:

(13) $$\begin{array}{c} \bar{G\circ H}=\bar{G}\circ \bar{H}. \end{array}$$

Consequently, the lexicographic product of two self-complementary graphs is itself self-complementary. Lexicographic products and their structural properties are further studied in Chapter 10 of [32].

The Shannon capacity of a graph, which is the main subject of this paper, is defined in terms of strong graph powers.

**Definition** **2** (Strong product)**.** *Let G and H be finite simple graphs. The strong product of G and H, denoted by G⊠H, is the graph with vertex set*

(14) $$\begin{array}{c} V(G⊠H)=V(G)\times V(H), \end{array}$$

*in which two distinct vertices (g,h) and $\left(g^{\prime },h^{\prime }\right)$ are adjacent if and only if*

(15) $$\begin{array}{c} \;\left(g=g^{\prime }\;\wedge \;h{∼}_{H}h^{\prime }\right)\;\vee \;\left(g{∼}_{G}g^{\prime }\;\wedge \;h=h^{\prime }\right)\;\vee \;\left(g{∼}_{G}g^{\prime }\;\wedge \;h{∼}_{H}h^{\prime }\right). \end{array}$$

*Equivalently, if $\left(g,h\right)\neq \left(g^{\prime },h^{\prime }\right)$, then*

(16) $$\begin{array}{c} \;\left(g,h\right){∼}_{G⊠H}\left(g^{\prime },h^{\prime }\right)\;⟺\;\left(g=g^{\prime }\;\vee \;g{∼}_{G}g^{\prime }\right)\;\wedge \;\left(h=h^{\prime }\;\vee \;h{∼}_{H}h^{\prime }\right). \end{array}$$

*For a graph G and an integer n≥1, the n-fold strong power of G, denoted by $G^{⊠n}$, is the strong product of n copies of G.*

The two graph products in Definitions 1 and 2 have the same vertex set, but their adjacency relations differ: if $g{∼}_{G}g^{\prime }$, then the lexicographic product places no restriction on *h* and $h^{\prime }$, whereas the strong product requires that $h=h^{\prime }$ or $h{∼}_{H}h^{\prime }$. This observation is formalized in the following lemma, which is used later in this paper.

**Lemma** **1.** *Let G and H be finite simple graphs. Then, the strong product G⊠H is a spanning subgraph of the lexicographic product G∘H.*

**Proof.**  Both G⊠H and G∘H have the same vertex set V(G)×V(H). It therefore suffices to prove that

(17) $$\begin{array}{c} E(G⊠H)\subseteq E(G\circ H). \end{array}$$

Recall that two distinct vertices (g,h) and $\left(g^{\prime },h^{\prime }\right)$ are adjacent in G∘H if and only if

$$\left\{g,g^{\prime }\right\}\in E\left(G\right)\;\vee \;\left(g=g^{\prime }\;\wedge \;\left\{h,h^{\prime }\right\}\in E\left(H\right)\right).$$

Let (g,h) and $\left(g^{\prime },h^{\prime }\right)$ be adjacent in G⊠H. By the definition of the strong product, one of the following holds:

$$\begin{array}{cccccc} & g=g^{\prime } & & \wedge & & \left\{h,h^{\prime }\right\}\in E\left(H\right),\\ & \left\{g,g^{\prime }\right\}\in E\left(G\right) & & \wedge & & h=h^{\prime },\\ & \left\{g,g^{\prime }\right\}\in E\left(G\right) & & \wedge & & \left\{h,h^{\prime }\right\}\in E\left(H\right). \end{array}$$

In the first case, (g,h) and $\left(g^{\prime },h^{\prime }\right)$ are adjacent in G∘H because $g=g^{\prime }$ and $\left\{h,h^{\prime }\right\}\in E\left(H\right)$. In the other two cases, they are adjacent in G∘H because $\left\{g,g^{\prime }\right\}\in E\left(G\right)$. Consequently, (17) holds. Since the two products have the same vertex set, G⊠H is a spanning subgraph of G∘H. □

**Proposition** **1.** *Let G and H be finite simple graphs. Then, the independence and clique numbers of their lexicographic product satisfy*

(18) $$\begin{array}{cc} \alpha (G\circ H) & =\alpha (G)\;\alpha (H), \end{array}$$

(19) $$\begin{array}{cc} \omega (G\circ H) & =\omega (G)\;\omega (H). \end{array}$$

For completeness, we prove Proposition 1. The independence-number identity (18) was proved in [33] and is also stated in Problem 27.1 of [32], where the corresponding clique-number identity (19) is not mentioned. We include a reformulation of the proof of (18), together with a proof of (19).

**Proof.**  We first prove (18). Let *A* and *B* be maximum independent sets in *G* and *H*, respectively. We claim that A×B is an independent set in G∘H. Indeed, consider two distinct vertices $\left(g,h\right),\left(g^{\prime },h^{\prime }\right)\in A\times B$. If $g\neq g^{\prime }$, then $g{≁}_{G}g^{\prime }$ because *A* is independent. If $g=g^{\prime }$, then $h{≁}_{H}h^{\prime }$ because *B* is independent. Thus, the two vertices are not adjacent in G∘H, and consequently

α(G∘H)≥|A×B|=α(G)α(H).

For the reverse inequality, let *I* be an independent set in G∘H, and define

$$I_{g}\left\{h\in V\left(H\right):\left(g,h\right)\in I\right\},\;g\in V\left(G\right).$$

Each $I_{g}$ is an independent set in *H*, and therefore $|I_{g}≔|\leq \alpha \left(H\right).$ Moreover, the set

$$P≔\{g\in V\left(G\right):I_{g}\neq ⌀\}$$

is independent in *G*. Indeed, if distinct $g,g^{\prime }\in P$ were adjacent in *G*, then every vertex of $\left\{g\right\}\times I_{g}$ would be adjacent in G∘H to every vertex of $\left\{g^{\prime }\right\}\times I_{g^{\prime }}$, contradicting the independence of *I*. Hence, |P|≤α(G), and

$$\left|I\right|=\sum_{g\in P}|I_{g}|\leq |P|\;\alpha \left(H\right)\leq \alpha \left(G\right)\;\alpha \left(H\right).$$

Taking the maximum over all independent sets *I* proves the reverse inequality

α(G∘H)≤α(G)α(H),

which then establishes equality (18).

We next prove (19). Combining identities (13) and (18) yields

(20) $$\begin{array}{cc} \omega (G\circ H) & =\alpha (\bar{G\circ H})\\ \; & =\alpha (\bar{G}\circ \bar{H})\\ \; & =\alpha \left(\bar{G}\right)\;\alpha \left(\bar{H}\right)\\ \; & =\omega (G)\;\omega (H), \end{array}$$

where the first and last equalities follow from the relation $\alpha \left(F\right)=\omega \left(\bar{F}\right)$, valid for every graph *F*. □

**Remark** **1.** *In contrast to Proposition 1, the independence number is, in general, only supermultiplicative under the strong product:*

α(G⊠H)≥α(G)α(H).

*Indeed, the Cartesian product of an independent set in G and an independent set in H is independent in G⊠H, whereas the inequality can be strict. For example, label the vertices of $C_{5}$ by the elements of $Z_{5}$, with two vertices adjacent when their difference is ±1 modulo 5. Then,*

$$S≔\left\{\left(i,2i\right):i\in Z_{5}\right\}=\left\{(0,0),(1,2),(2,4),(3,1),(4,3)\right\}$$

*is an independent set in $C_{5}⊠C_{5}$. In fact,*

$$5=\alpha \left(C_{5}⊠C_{5}\right)>\alpha {\left(C_{5}\right)}^{2}=4.$$

*The clique number, on the other hand, is multiplicative under the strong product:*

ω(G⊠H)=ω(G)ω(H).

*To see this, let A and B be maximum cliques in G and H, respectively. For any two distinct vertices $\left(g,h\right),\left(g^{\prime },h^{\prime }\right)\in A\times B$, we have*

$$g=g^{\prime }\;\mathrm{or}\;g{∼}_{G}g^{\prime },\;h=h^{\prime }\;\mathrm{or}\;h{∼}_{H}h^{\prime }.$$

*Thus, A×B is a clique in G⊠H, and hence ω(G⊠H)≥|A×B|=ω(G)ω(H). For the reverse inequality, let C be a clique in G⊠H, and let*

$$\begin{array}{cc} \; & P_{G}≔\left\{g\in V(G):(g,h)\in C\;\mathrm{for}\;\mathrm{some}\;h\in V(H)\right\},\\ \; & P_{H}≔\left\{h\in V(H):(g,h)\in C\;\mathrm{for}\;\mathrm{some}\;g\in V(G)\right\} \end{array}$$

*be its coordinate projections. The set $P_{G}$ is a clique in G. Indeed, if $g,g^{\prime }\in P_{G}$ are distinct, then there exist $h,h^{\prime }\in V\left(H\right)$ such that $\left(g,h\right),\left(g^{\prime },h^{\prime }\right)\in C$. Since these vertices are adjacent in G⊠H and $g\neq g^{\prime }$, it follows that $g{∼}_{G}g^{\prime }$. Similarly, $P_{H}$ is a clique in H. Therefore,*

$$|P_{G}\left|\leq \omega \left(G\right),\;\right|P_{H}|\leq \omega \left(H\right).$$

*Since $C\subseteq P_{G}\times P_{H}$, we obtain*

$$\left|C|\leq \right|P_{G}|\;|P_{H}|\leq \omega \left(G\right)\;\omega \left(H\right).$$

*Maximizing over all cliques C gives the reverse inequality and proves the claimed equality.*

*Thus, the distinction between the two products lies in the behavior of the independence number: it is multiplicative under the lexicographic product, but in general only supermultiplicative under the strong product.*

**Remark** **2.** *Although it follows from Proposition 1 that*

α(G∘H)=α(H∘G)andω(G∘H)=ω(H∘G),

*the graphs G∘H and H∘G need not be isomorphic. In contrast, the strong product is commutative up to graph isomorphism:*

G⊠H≅H⊠G.

*Indeed, the coordinate-switching map*

V(G⊠H)⟶V(H⊠G),(g,h)⟼(h,g),

*is a graph isomorphism.*

### 2.2. Lovász Theta Function

The Lovász ϑ-function is a graph invariant introduced in [6] and defined in terms of orthogonal representations of the graph. It can be computed efficiently and provides computable bounds on several graph invariants whose exact computation is NP-hard. It has been studied, e.g., in [5,6,8,10,18,25]. The Lovász ϑ-function of a finite simple graph *G* can be expressed as a solution of a semidefinite programming (SDP) problem. To that end, let $A=(A_{i,j})$ be the n×n adjacency matrix of *G* with n≜|V(G)|. The Lovász ϑ-function ϑ(G) can be expressed by the following convex optimization problem:

(21) maximizeTr(BJn)subjecttoB⪰0,Tr(B)=1,Ai,j=1⇒Bi,j=0,i,j∈[n].

The SDP formulation in (21) yields the existence of an algorithm that computes ϑ(G), for every graph *G*, with a precision of *r* decimal digits, and a computational complexity that is polynomial in *n* and *r*. Thus, the Lovász ϑ-function can be computed in polynomial time in *n*, and by [6], it is an upper bound on the Shannon capacity. By contrast, determining the Shannon capacity involves an infinite sequence of independence numbers, each of which is NP-hard to compute.

### 2.3. Fractional Haemers Number

The fractional Haemers number is a graph invariant studied in [16,30,34,35,36]. We provide here some essential background.

**Definition** **3** (Fractional Haemers number)**.** *Let G=(V,E) be a finite simple graph, let F be a field, and write [d]≔{1,…,d} for an integer d≥1. A matrix*

$$M\in F^{\left(V\times [d])\times (V\times [d]\right)}$$

*is called a d-representation of G over* F *if its d×d blocks $M_{u,v}$, indexed by u,v∈V, satisfy*

$$M_{v,v}=I_{d}\;\mathrm{for}\;\mathrm{every}\;v\in V,$$

*and*

$$M_{u,v}=M_{v,u}=O_{d}\;\mathrm{whenever}\;u\neq v\;\mathrm{and}\;\left\{u,v\right\}\notin E,$$

*where $I_{d}$ and $O_{d}$ denote the d×d identity and zero matrices, respectively. For an integer d≥1, let $M_{d}\left(G;F\right)$ denote the set of all d-representations of G over F.*

*1.*  *The fractional Haemers number of G over F is defined by*
(22) $$\begin{array}{c} H_{f}\left(G;F\right)≔\underset{d\geq 1}{\inf}\;\min_{M\in M_{d}\left(G;F\right)}\frac{{\mathrm{rank}}_{F}\left(M\right)}{d}. \end{array}$$
*2.*  *Restricting the optimization to d=1 gives the (ordinary) Haemers number [15], defined by*
(23) $$\begin{array}{c} H\left(G;F\right)≔\min_{M\in M_{1}\left(G;F\right)}{\mathrm{rank}}_{F}\left(M\right). \end{array}$$

**Definition** **4** (Fractional Lovász theta function)**.** *Let G be a finite simple graph. The fractional Lovász theta function of G is defined by*

(24) $$\begin{array}{c} \vartheta_{f}\left(G\right)≔\underset{d\geq 1}{\inf}\frac{\vartheta \left(G\circ \bar{K_{d}}\right)}{d}, \end{array}$$

*where $\bar{K_{d}}$ is the edgeless graph on d vertices.*

**Proposition** **2.** *Let G be a finite simple graph, and let F be a field. Then,*

(25) $$\begin{array}{c} H_{f}\left(G;F\right)=\underset{d\geq 1}{\inf}\frac{H\left(G\circ \bar{K_{d}};F\right)}{d}, \end{array}$$

*where $\bar{K_{d}}$ denotes the edgeless graph on d vertices.*

**Proof.**  For every integer d≥1, the vertex set of $G\circ \bar{K_{d}}$ is V(G)×[d]. Two distinct vertices (u,i) and (v,j) are nonadjacent in $G\circ \bar{K_{d}}$ precisely when either u=v, or u≠v and {u,v}∉E(G). Consequently, a matrix fitting $G\circ \bar{K_{d}}$ over F, viewed as a block matrix whose d×d blocks are indexed by u,v∈V(G), has diagonal blocks equal to $I_{d}$ and zero blocks whenever u≠v and {u,v}∉E(G). Thus, the matrices fitting $G\circ \bar{K_{d}}$ are precisely the *d*-representations of *G* over F. It follows that

(26) $$\begin{array}{c} H\left(G\circ \bar{K_{d}};F\right)=\min_{M\in M_{d}\left(G;F\right)}{\mathrm{rank}}_{F}\left(M\right). \end{array}$$

Dividing both sides of (26) by *d* and taking the infimum over all integers d≥1, we obtain (25) by (22). □

### 2.4. Shannon Capacity of Graphs

**Definition** **5** (Shannon capacity of a graph)**.** *The Shannon capacity of a finite simple graph G is defined as*

(27) $$\begin{array}{c} \Theta \left(G\right)≔\underset{n\geq 1}{\sup}\sqrt[{n}]{\alpha \;\left(G^{⊠n}\right)}=\lim_{n\to \infty }\sqrt[{n}]{\alpha \;\left(G^{⊠n}\right)}, \end{array}$$

*where the existence of the limit and the last equality follow from Fekete’s lemma and the supermultiplicativity of the independence number under strong products.*

The following result by Lovász provides an easy-to-compute upper bound on the Shannon capacity of graphs (see Theorem 1 of [6]).

**Theorem** **1.** *Let G be a finite simple graph. Then, its Shannon capacity is upper bounded by the Lovász theta function:*

(28) $$\begin{array}{c} \Theta (G)\leq \vartheta (G). \end{array}$$

The next upper bound on the Shannon capacity was first proved by Blasiak [34]; see also Theorem 1 of [16].

**Theorem** **2.** *Let G be a finite simple graph, and let F be a field. Then, the Shannon capacity of G is upper bounded by its fractional Haemers number over F; that is,*

(29) $$\begin{array}{c} \Theta \left(G\right)\leq H_{f}\left(G;F\right). \end{array}$$

*Moreover,*

(30) $$\begin{array}{c} \alpha \left(G\right)\leq \Theta \left(G\right)\leq H_{f}\left(G;F\right)\leq H\left(G;F\right). \end{array}$$

**Proof.**  For every finite graph *K*,

$$\alpha \left(K\right)\leq H_{f}\left(K;F\right).$$

Applying this inequality to the *n*-th strong power of *G*, and using the multiplicativity of the fractional Haemers number under the strong product (see Theorem 3 of [16]), gives

$$\alpha \left(G^{⊠n}\right)\leq H_{f}\left(G^{⊠n};F\right)=H_{f}{\left(G;F\right)}^{n}.$$

Taking *n*-th roots and then the supremum over n≥1, we obtain

$$\Theta \left(G\right)=\underset{n\geq 1}{\sup}\sqrt[{n}]{\alpha \left(G^{⊠n}\right)}\leq H_{f}\left(G;F\right).$$

Finally, restricting the definition of $H_{f}\left(G;F\right)$ to d=1 yields

$$H_{f}\left(G;F\right)\leq H\left(G;F\right),$$

while α(G)≤Θ(G) follows by taking n=1 in the middle term of (27). □

In general, the fractional Haemers number and the Lovász theta function are incomparable. That is, depending on the graph and the underlying field, either one may provide the stronger upper bound on the Shannon capacity. In particular, Bukh and Cox established the following strict separation.

**Theorem** **3** (Bukh and Cox, Theorem 2 of [16])**.** *Let F be a field of nonzero characteristic. Then, there exists a finite graph G=G(F) such that, for every field $F^{\prime }$,*

(31) $$\begin{array}{c} H_{f}\left(G;F\right)<\min\left\{H\left(G;F^{\prime }\right),\vartheta \left(G\right)\right\}. \end{array}$$

*In particular,*

(32) $$\begin{array}{c} H_{f}\left(G;F\right)<\vartheta \left(G\right). \end{array}$$

A further explicit separation was obtained by Hu, Tamo, and Shayevitz (see Example 6 and Remark 3 of [17]). Let *S* denote the complement of the Schläfli graph, and let

(33) $$\begin{array}{c} G≔S+7C_{5}, \end{array}$$

where + denotes the disjoint union. They showed that

$$H_{f}\left(G;F_{11}\right)\leq \frac{49}{2}<9+7\sqrt{5}=\vartheta \left(G\right)<28=H\left(G\right),$$

where

(34) $$\begin{array}{c} H\left(G\right)≔\min_{F^{\prime }}H\left(G;F^{\prime }\right) \end{array}$$

is the field-independent Haemers number. Thus, this graph satisfies

(35) $$\begin{array}{c} H_{f}\left(G;F_{11}\right)<\vartheta \left(G\right)<H\left(G\right). \end{array}$$

### 2.5. Kneser Graphs

Kneser graphs form an important family of highly symmetric graphs in algebraic and extremal graph theory.

**Definition** **6.** *The Kneser graph KG(n,k), defined for integers n≥2k, has as its vertices all k-element subsets of [n]≔{1,…,n}, with two vertices adjacent if and only if the corresponding subsets are disjoint.*

By Definition 6, KG(n,k) has nk vertices and is n−kk-regular. Independent sets in KG(n,k) correspond precisely to intersecting families of *k*-element subsets of [n]. Consequently, the Erdős–Ko–Rado theorem gives

(36) αKG(n,k)=n−1k−1,n≥2k.

Moreover, the Shannon capacity of KG(n,k) equals its independence number (see Theorem 13 of [6]), and hence

(37) ΘKG(n,k)=n−1k−1,n≥2k.

**Example** **2.** *The Kneser graph KG(5,2) is isomorphic to the well-known Petersen graph. Its vertices are the ten 2-element subsets of [5], with two vertices adjacent if and only if the corresponding subsets are disjoint. For example, the vertex {1,2} is adjacent precisely to {3,4}, {3,5}, and {4,5}. Thus, KG(5,2) is a 3-regular graph on 10 vertices. By *(36)*, its independence number is 4; for example, $\left\{\{1,2\},\{1,3\},\{1,4\},\{1,5\}\right\}$ is a maximum independent set. Moreover, by *(37)*, its Shannon capacity is also equal to* 4*, that is,*

$$\Theta \left(KG(5,2)\right)=\alpha \left(KG(5,2)\right)=4.$$

We next define *q*-analogues of Kneser graphs and Gaussian binomial coefficients; the interested reader is referred to Chapter 9 of [37] for more information.

**Definition** **7.** *Let n,k,m≥1 be integers with k≤n, let p be a prime, and let $q≔p^{m}$. Let $F_{q}$ denote the finite field of order q. The Gaussian binomial coefficient, denoted by ${\left[\;\begin{array}{c} \;n\\ \;k\; \end{array}\right]}_{q}$, is defined as*

(38) $$\begin{array}{c} {\left[\begin{array}{c} n\\ k \end{array}\right]}_{q}≔\frac{\left(q^{n}-1\right)\left(q^{n}-q\right)\cdots \left(q^{n}-q^{k-1}\right)}{\left(q^{k}-1\right)\left(q^{k}-q\right)\cdots \left(q^{k}-q^{k-1}\right)}. \end{array}$$

The Gaussian coefficient admits the following combinatorial interpretation. Let *V* be an *n*-dimensional vector space over $F_{q}$. Then, the number of distinct *k*-dimensional subspaces of *V* is equal to ${\left[\;\begin{array}{c} \;n\\ \;k\; \end{array}\right]}_{q}$. Allowing *q* to vary continuously and taking q→1 gives

(39) limq→1nkq=nk·n−1k−1⋯n−(k−1)k−(k−1)=nk,

thus converging to the binomial coefficient.

**Definition** **8** (*q*-Kneser graphs)**.** *Let V(n,q) denote the n-dimensional vector space over the finite field $F_{q}$, where q is a prime power. The q-Kneser graph ${\mathrm{KG}}_{q}\left(n,k\right)$ has as its vertices the k-dimensional subspaces of V(n,q), and any two vertices are adjacent if and only if their intersection is the zero subspace.*

By Theorem 6.6 of [5],
  (40) $$\begin{array}{c} \Theta \left({\mathrm{KG}}_{q}\left(n,k\right)\right)=\alpha \left({\mathrm{KG}}_{q}\left(n,k\right)\right)={\left[\;\begin{array}{c} \;n-1\\ \;k-1\; \end{array}\right]}_{q},\;n\geq 2k. \end{array}$$

### 2.6. Paley Graphs

**Definition** **9.** *Let n≥5 be a prime power such that n≡1(mod4). The Paley graph P(n) is the graph with vertex set $V\left(P\left(n\right)\right)=F_{n},$ in which two distinct vertices $x,y\in F_{n}$ are adjacent if and only if x−y is a nonzero square in $F_{n}$.*

The congruence condition n≡1(mod4) ensures that −1 is a square in $F_{n}$. Hence, x−y is a square if and only if y−x is a square, so the adjacency relation is symmetric. Paley graphs are self-complementary, vertex-transitive, and strongly regular. More precisely, P(n) has parameters

$$\mathrm{srg}\left(n,\frac{n-1}{2},\frac{n-5}{4},\frac{n-1}{4}\right).$$

Thus, P(n) is a regular graph on *n* vertices of degree $\frac{n-1}{2}$; every pair of adjacent vertices has exactly $\lambda =\frac{n-5}{4}$ common neighbors, whereas every pair of nonadjacent vertices has exactly $\mu =\frac{n-1}{4}$ common neighbors.

**Example** **3.** *The smallest Paley graph, P(5), is isomorphic to the cycle $C_{5}$. Indeed, the nonzero squares in $F_{5}=\left\{0,1,2,3,4\right\}$ are 1 and 4=−1. Hence, two distinct vertices $x,y\in F_{5}$ are adjacent if and only if x−y≡±1(mod5), which gives the 5-length cycle 0−1−2−3−4−0. Accordingly, P(5) is self-complementary and vertex-transitive, and it is strongly regular with parameters srg(5,2,0,1). In particular, every pair of adjacent vertices has no common neighbor, whereas every pair of nonadjacent vertices has exactly one common neighbor. Moreover, P(5) is the unique triangle-free Paley graph: for every admissible n>5, each pair of adjacent vertices in P(n) has $\lambda =\frac{n-5}{4}\geq 1$ common neighbors and therefore lies in a triangle.*

## 3. Lovász Theta Function for Lexicographic Graph Products

The next result was established in greater generality in Theorem 8.2 of [30]. We provide an alternative simplified proof for the specialized setting considered here.

**Theorem** **4.** *For all finite simple graphs G and H,*

(41) $$\begin{array}{c} \vartheta (G\circ H)=\vartheta (G)\;\vartheta (H). \end{array}$$

**Proof.**  We prove separately the two inequalities that give equality (41).

**Lower bound.** We use the semidefinite-programming formulation (21) of the Lovász ϑ-function:
  $$\vartheta \left(G\right)=\max\left\{\langle J,X\rangle :X⪰0,\;\mathrm{Tr}\left(X\right)=1,\;X_{g,g^{\prime }}=0\;\mathrm{whenever}\;g{∼}_{G}g^{\prime }\right\},$$
  where *J* is the all-ones matrix, and $\langle J,X\rangle =\mathrm{Tr}\left(JX\right)$.

Let *X* and *Y* be optimal solutions to the semidefinite programs defining ϑ(G) and ϑ(H), respectively, and let

Z≔X⊗Y.

Then,

Z⪰0,Tr(Z)=Tr(X)Tr(Y)=1.

If $\left(g,h\right){∼}_{G\circ H}\left(g^{\prime },h^{\prime }\right)$, then either $g{∼}_{G}g^{\prime }$, in which case $X_{g,g^{\prime }}=0$, or $g=g^{\prime }$ and $h{∼}_{H}h^{\prime }$, in which case $Y_{h,h^{\prime }}=0$. Consequently,

$$Z_{\left(g,h\right),\left(g^{\prime },h^{\prime }\right)}=X_{g,g^{\prime }}\;Y_{h,h^{\prime }}=0.$$

Therefore, *Z* is feasible for the semidefinite program (21) defining ϑ(G∘H):

(42) $$\begin{array}{cc} \vartheta (G\circ H)=\max\{\; & \langle J,Z\rangle :Z⪰0,\;\mathrm{Tr}\left(Z\right)=1,\\ \; & Z_{\left(g,h\right),\left(g^{\prime },h^{\prime }\right)}=0\;\mathrm{whenever}\;\;\left(g,h\right){∼}_{G\circ H}\left(g^{\prime },h^{\prime }\right)\;\}. \end{array}$$

Moreover, since

$$J_{\left|V(G)|\;|V(H)\right|}=J_{\left|V(G)\right|}⊗J_{\left|V(H)\right|},$$

the standard identities for Kronecker products give

(43) $$\begin{array}{cc} \langle J,Z\rangle & =\mathrm{Tr}\left(\left(J_{\left|V(G)\right|}⊗J_{\left|V(H)\right|}\right)\left(X⊗Y\right)\right)\\ \; & =\mathrm{Tr}\left(\left(J_{\left|V(G)\right|}X\right)⊗\left(J_{\left|V(H)\right|}Y\right)\right)\\ \; & =\mathrm{Tr}\left(J_{\left|V(G)\right|}X\right)\;\mathrm{Tr}\left(J_{\left|V(H)\right|}Y\right)\\ \; & =\langle J,X\rangle \langle J,Y\rangle \\ \; & =\vartheta (G)\;\vartheta (H), \end{array}$$

which, by (42) and (43), implies that

(44) $$\begin{array}{c} \vartheta (G\circ H)\geq \vartheta (G)\;\vartheta (H). \end{array}$$

**Upper bound.** By Lemma 1, G⊠H is a spanning subgraph of G∘H. Since the Lovász theta function is monotonically nonincreasing under the addition of edges, it follows that

ϑ(G∘H)≤ϑ(G⊠H).

Combining the last inequality with the identity ϑ(G⊠H)=ϑ(G)ϑ(H) (see Theorem 7 of [6]) gives

(45) $$\begin{array}{c} \vartheta (G\circ H)\leq \vartheta (G)\;\vartheta (H). \end{array}$$

Combining inequalities (44) and (45) proves equality (41). □

**Corollary** **1.** *For all integers m≥1, the m-fold join of G with itself satisfies*

(46) $$\begin{array}{c} \vartheta \left(K_{m}\circ G\right)=\vartheta \left(G\right). \end{array}$$

**Proof.**  This holds by Theorem 4 and since $\vartheta (K_{m})=1$. □

Applying Theorem 4 yields the following result from Theorem 20 of [16], asserting the equality of the fractional and ordinary Lovász theta functions, together with a simplified proof.

**Corollary** **2.** *Let G be a finite simple graph. Then,*

(47) $$\begin{array}{c} \vartheta_{f}\left(G\right)=\vartheta \left(G\right). \end{array}$$

**Proof.**  By Definition 4, Theorem 4, and the identity $\vartheta (\bar{K_{d}})=d,$ where $\bar{K_{d}}$ is the edgeless graph on *d* vertices, we have

$$\begin{array}{cc} \vartheta_{f}\left(G\right) & =\underset{d\geq 1}{\inf}\frac{\vartheta \left(G\circ \bar{K_{d}}\right)}{d}\\ \; & =\underset{d\geq 1}{\inf}\frac{\vartheta \left(G\right)\;\vartheta \left(\bar{K_{d}}\right)}{d}\\ \; & =\vartheta (G). \end{array}$$

This proves (47). □

## 4. Fractional Haemers Number for Lexicographic Graph Products

The following result establishes the multiplicativity of the fractional Haemers number under the lexicographic product. It was stated by Fritz in Proposition 4.13 of [36], where the lexicographic-product argument is credited to Cox and described as an adaptation of the argument of Bukh and Cox [16]. The proof presented there is different and relies on previously established results. We next give an alternative, self-contained proof.

**Theorem** **5.** *Let G and H be finite simple graphs, and let F be a field. Then,*

(48) $$\begin{array}{c} H_{f}\left(G\circ H;F\right)=H_{f}\left(G;F\right)\;H_{f}\left(H;F\right). \end{array}$$

**Proof.**  We prove equality (48) by establishing the two corresponding inequalities separately.

**Upper bound.** We first prove an upper bound on $H_{f}\left(G\circ H;F\right)$. Let d,e≥1 be arbitrary integers, let *M* be a *d*-representation of *G* over F, and let *N* be an *e*-representation of *H* over F. Define a block matrix *P*, indexed by V(G)×V(H), by

$$P_{\left(u,x),(v,y\right)}≔M_{u,v}⊗N_{x,y},\;\mathrm{for}\;\mathrm{all}\;u,v\in V\left(G\right)\;\mathrm{and}\;x,y\in V\left(H\right).$$

Each block of *P* has size de×de. Moreover, for every u∈V(G) and x∈V(H),

$$P_{\left(u,x),(u,x\right)}=M_{u,u}⊗N_{x,x}=I_{d}⊗I_{e}=I_{de}.$$

Suppose that (u,x) and (v,y) are distinct and nonadjacent in G∘H. Then, either

u≠vand{u,v}∉E(G),

or

u=vand{x,y}∉E(H).

In the first case, $M_{u,v}=O_{d}$, and in the second case, $N_{x,y}=O_{e}$. Hence,

$$P_{\left(u,x),(v,y\right)}=O_{de}.$$

Thus, *P* is a de-representation of G∘H. After suitable permutations of its scalar rows and columns, *P* is equal to the Kronecker product M⊗N. Since such permutations preserve rank,

$$\begin{array}{cc} {\mathrm{rank}}_{F}\left(P\right) & ={\mathrm{rank}}_{F}\left(M⊗N\right)\\ \; & ={\mathrm{rank}}_{F}\left(M\right)\;{\mathrm{rank}}_{F}\left(N\right), \end{array}$$

and consequently, since *P* is a de-representation of G∘H, the definition of the fractional Haemers number gives

$$H_{f}\left(G\circ H;F\right)\leq \frac{{\mathrm{rank}}_{F}\left(P\right)}{de}=\frac{{\mathrm{rank}}_{F}\left(M\right)}{d}\;\frac{{\mathrm{rank}}_{F}\left(N\right)}{e}.$$

This inequality holds for every *d*-representation *M* of *G* and every *e*-representation *N* of *H* over F. Since these choices are independent, taking the infimum of the rightmost term over all integers d,e≥1 and all corresponding representations *M* and *N*, we obtain

(49) $$\begin{array}{c} H_{f}\left(G\circ H;F\right)\leq H_{f}\left(G;F\right)\;H_{f}\left(H;F\right). \end{array}$$

**Reverse inequality.** We next prove the reverse inequality. The argument consists of four steps. Starting from an arbitrary *k*-representation *P* of G∘H, we first restrict *P* to the copies of *H*. We then compress these restrictions to a common dimension *r* and use them to construct an *r*-representation of *G*. Finally, we combine the resulting bounds for *G* and *H*.

**Step 1: Restriction to the copies of** *H***.** Let *k* be an arbitrary positive integer and let *P* be a *k*-representation of G∘H over F. Set
  (50) $$\begin{array}{c} n≔{\mathrm{rank}}_{F}\left(P\right), \end{array}$$
  and choose a rank factorization
  (51) $$\begin{array}{c} P=AB, \end{array}$$
  where *A* has *n* columns and *B* has *n* rows.
The matrix *P* is a block matrix indexed by V(G)×V(H), with each block having size k×k. Thus, its scalar rows and columns may be indexed by
  V(G)×V(H)×[k],[k]≔{1,…,k}.
  The rows of *A* and the columns of *B* inherit the corresponding scalar indices from the rank factorization P=AB.
For each u∈V(G), let $A_{u}$ be the submatrix of *A* whose rows are indexed by

{u}×V(H)×[k],

and let $B_{u}$ be the submatrix of *B* whose columns are indexed by the same set. Then,

(52) $$\begin{array}{c} P_{u}≔A_{u}B_{u} \end{array}$$

is the principal submatrix of *P* corresponding to {u}×V(H). In particular, $P_{u}$ is a *k*-representation of *H*. Define

(53) $$\begin{array}{c} r_{u}≔{\mathrm{rank}}_{F}\left(P_{u}\right),\;r≔\min_{u\in V(G)}r_{u}. \end{array}$$

Since $P_{u}$ contains an identity matrix $I_{k}$ as a diagonal block, we have $r_{u}\geq k$ for every u∈V(G). In particular, by (53),

r≥k>0.

Since $P_{u}$ is a *k*-representation of *H* over F, from (22) and (53), for every u∈V(G) we have

$$\frac{r_{u}}{k}\geq H_{f}\left(H;F\right).$$

Taking the minimum over u∈V(G), we obtain

(54) $$\begin{array}{c} \frac{r}{k}\geq H_{f}\left(H;F\right). \end{array}$$

**Step 2: Compression to the common dimension** *r***.** We next prepare the construction of an *r*-representation of *G*. For each u∈V(G), we have, by (52) and (53),

(55) $$\begin{array}{c} {\mathrm{rank}}_{F}\left(A_{u}B_{u}\right)=r_{u}\geq r. \end{array}$$

Consequently, $A_{u}B_{u}$ contains a nonsingular r×r submatrix. Hence, there exist binary row- and column-selection matrices

$$R_{u}\in F^{\;r\times k|V(H)|},\;S_{u}\in F^{\;k|V(H)|\times r},$$

respectively, such that

(56) $$\begin{array}{c} T_{u}≔R_{u}A_{u}B_{u}S_{u} \end{array}$$

is nonsingular. Define

(57) $$\begin{array}{c} X_{u}≔T_{u}^{-1}R_{u},\;Y_{u}≔S_{u}. \end{array}$$

Then, by (56) and (57),

(58) $$\begin{array}{c} X_{u}A_{u}B_{u}Y_{u}=T_{u}^{-1}R_{u}A_{u}B_{u}S_{u}=T_{u}^{-1}T_{u}=I_{r}. \end{array}$$

Upon setting

(59) $$\begin{array}{c} C_{u}≔X_{u}A_{u}\in F^{r\times n},\;D_{u}≔B_{u}Y_{u}\in F^{n\times r}, \end{array}$$

it follows from (58) and (59) that

(60) $$\begin{array}{c} C_{u}D_{u}=X_{u}A_{u}B_{u}Y_{u}=I_{r}. \end{array}$$

**Step 3: Construction of an** *r***-representation of** *G***.** Define a block matrix *Q*, indexed by V(G), by

(61) $$\begin{array}{c} Q_{u,v}≔C_{u}D_{v}. \end{array}$$

By (60), its diagonal blocks satisfy

(62) $$\begin{array}{c} Q_{u,u}=C_{u}D_{u}=I_{r}. \end{array}$$

Now, suppose that u≠v and {u,v}∉E(G). Every vertex in the copy of *H* corresponding to *u* is then nonadjacent in G∘H to every vertex in the copy corresponding to *v*. Hence, the corresponding submatrix of *P* is zero, that is,

(63) $$\begin{array}{c} A_{u}B_{v}=O_{k\;|V(H)|}, \end{array}$$

and since $C_{u}=X_{u}A_{u}$ and $D_{v}=B_{v}Y_{v}$ (see (59)), we obtain

(64) $$\begin{array}{c} C_{u}D_{v}=X_{u}A_{u}B_{v}Y_{v}=O_{r}. \end{array}$$

Thus, *Q* is an *r*-representation of *G* over F.

It remains to relate the rank of *Q* to that of *P*. Fix an ordering $V\left(G\right)=\left\{u_{1},\ldots ,u_{m}\right\}$, and define

(65) $$\hat{C}≔\left(\begin{array}{c} C_{u_{1}}\\ \vdots \\ C_{u_{m}} \end{array}\right),\;\hat{D}≔\left(\begin{array}{ccc} D_{u_{1}} & \cdots & D_{u_{m}} \end{array}\right).$$

By the definition of the blocks of *Q*,

(66) $$\begin{array}{c} Q=\hat{C}\hat{D}. \end{array}$$

Since $\hat{C}$ has *n* columns and $\hat{D}$ has *n* rows, it follows that

(67) $$\begin{array}{c} {\mathrm{rank}}_{F}\left(Q\right)\leq n, \end{array}$$

and therefore

(68) $$\begin{array}{c} H_{f}\left(G;F\right)\leq \frac{{\mathrm{rank}}_{F}\left(Q\right)}{r}\leq \frac{n}{r}. \end{array}$$

**Step 4: Completion of the proof.** Combining (50), (54), and (68) yields

(69) $$\begin{array}{c} H_{f}\left(G;F\right)\;H_{f}\left(H;F\right)\leq \frac{n}{r}\;\frac{r}{k}=\frac{{\mathrm{rank}}_{F}\left(P\right)}{k}. \end{array}$$

Since this holds for all integers k≥1 and all *k*-representations *P* of G∘H, taking the infimum of the rightmost term in (69) over all such *k* and *P* yields

(70) $$\begin{array}{c} H_{f}\left(G;F\right)\;H_{f}\left(H;F\right)\leq H_{f}\left(G\circ H;F\right). \end{array}$$

Combining inequalities (49) and (70) proves equality (48). □

## 5. Shannon Capacity for Lexicographic Graph Products

Our first result establishes a general comparison among the Shannon capacities of the two ordered lexicographic products, the strong product, and the individual factors. Since the lexicographic product is not commutative in general, both G∘H and H∘G must be considered. The strong product G⊠H is a spanning subgraph of G∘H, and, up to the natural interchange of coordinates, also of H∘G. Hence, the monotonicity of Shannon capacity with respect to the addition of edges gives an upper bound on the capacities of both lexicographic products. Together with a product construction for independent sets and the multiplicativity of the Lovász theta function under the strong product, this yields the following chain of bounds.

**Theorem** **6** (Bounds for the Shannon capacity)**.** *For all finite simple graphs G and H,*

(71) $$\begin{array}{cc} \Theta (G)\;\Theta (H) & \leq \min\{\Theta (G\circ H),\Theta (H\circ G)\}\\ \; & \leq \max\{\Theta (G\circ H),\Theta (H\circ G)\}\\ \; & \leq \Theta (G⊠H)\leq \vartheta (G)\;\vartheta (H). \end{array}$$

*In particular,*

(72) $$\begin{array}{cc} \;\Theta (G⊠H)=\Theta (G)\;\Theta (H) & \;⟹\;\Theta (G\circ H)=\Theta (G)\;\Theta (H), \end{array}$$

(73) $$\begin{array}{cc} \;\left(\Theta (G)=\vartheta (G),\;\Theta (H)=\vartheta (H)\right) & \;⟹\;\Theta (G\circ H)=\Theta (H\circ G)=\vartheta (G)\;\vartheta (H). \end{array}$$

**Proof.**  Since the strong product is commutative, whereas the lexicographic product is generally noncommutative (see Remark 2), it suffices to prove that

(74) $$\begin{array}{c} \Theta (G)\;\Theta (H)\leq \Theta (G\circ H)\leq \Theta (G⊠H)\leq \vartheta (G)\;\vartheta (H). \end{array}$$

Fix n≥1, and let

(75) $$\begin{array}{c} A\subseteq V\;\left(G^{⊠n}\right),\;B\subseteq V\;\left(H^{⊠n}\right) \end{array}$$

be maximum independent sets. Define the map

(76) $$\begin{array}{c} \Phi_{n}:V\;\left(G^{⊠n}\right)\times V\;\left(H^{⊠n}\right)⟶V\;\left({\left(G\circ H\right)}^{⊠n}\right) \end{array}$$

by

(77) $$\begin{array}{c} \Phi_{n}\left(g,h\right)≔\left(\left(g_{1},h_{1}\right),\ldots ,\left(g_{n},h_{n}\right)\right), \end{array}$$

where

$$g=\left(g_{1},\ldots ,g_{n}\right),\;h=\left(h_{1},\ldots ,h_{n}\right).$$

We show that

(78) $$\begin{array}{c} C≔\Phi_{n}\left(A\times B\right) \end{array}$$

is an independent set in ${\left(G\circ H\right)}^{⊠n}$. Indeed, consider two distinct elements $\Phi_{n}\left(g,h\right)$ and $\Phi_{n}\left(g^{\prime },h^{\prime }\right)$ of C. If $g\neq g^{\prime }$, then the independence of A implies that there exists a coordinate *j* such that $g_{j}\neq g_{j}^{\prime }$ and $g_{j}{≁}_{G}g_{j}^{\prime }$. It follows from the definition of the lexicographic product that $\left(g_{j},h_{j}\right)$ and $\left(g_{j}^{\prime },h_{j}^{\prime }\right)$ are nonadjacent in G∘H. Otherwise, if $g=g^{\prime }$, then $h\neq h^{\prime }$. The independence of B therefore implies that there exists a coordinate *j* such that $h_{j}\neq h_{j}^{\prime }$ and $h_{j}{≁}_{H}h_{j}^{\prime }$. Since $g_{j}=g_{j}^{\prime }$, the vertices $\left(g_{j},h_{j}\right)$ and $\left(g_{j}^{\prime },h_{j}^{\prime }\right)$ are again nonadjacent in G∘H. Thus, C is independent. Furthermore, since $\Phi_{n}$ is injective,

(79) $$\begin{array}{c} |C|=|A\times B|=|A|\;|B|. \end{array}$$

Since A and B are, by assumption, maximum independent sets in $G^{⊠n}$ and $H^{⊠n}$, respectively, and the set C defined in (78) is independent in ${\left(G\circ H\right)}^{⊠n}$ with cardinality given by (79), it follows that

(80) $$\begin{array}{c} \alpha \;\left({\left(G\circ H\right)}^{⊠n}\right)\geq \alpha \;\left(G^{⊠n}\right)\;\alpha \;\left(H^{⊠n}\right). \end{array}$$

Taking the *n*-th roots of both sides of (80) and letting n→∞, yields

(81) $$\begin{array}{c} \Theta (G\circ H)\geq \Theta (G)\;\Theta (H). \end{array}$$

By Lemma 1, G⊠H is a spanning subgraph of G∘H. Since Shannon capacity is nonincreasing under the addition of edges, it follows that

(82) $$\begin{array}{c} \Theta (G\circ H)\leq \Theta (G⊠H). \end{array}$$

Finally, by Theorems 1 and 7 of [6],

(83) $$\begin{array}{c} \Theta (G⊠H)\leq \vartheta (G⊠H)=\vartheta (G)\;\vartheta (H). \end{array}$$

Combining (81)–(83) completes the proof of (71). Finally, assertions (72) and (73) follow directly from (71). □

**Corollary** **3** (Shannon capacity of the lexicographic power of a graph)**.** *Let G be a finite, undirected, and simple graph, and let m≥1 be an integer. Then,*

(84) $$\begin{array}{c} \Theta \left(G^{\;\circ m}\right)=\Theta {\left(G\right)}^{m}, \end{array}$$

*where $G^{\;\circ m}$ denotes the m-fold lexicographic power of G.*

**Proof.**  By Theorem 6, it follows that for every integer m≥1

$$\begin{array}{cc} \Theta {\left(G\right)}^{m} & \leq \Theta (G^{\;\circ m})\\ \; & \leq \Theta (G^{\;⊠m})\\ \; & =\Theta {\left(G\right)}^{m}, \end{array}$$

thus proving (84). □

Theorem 6 states that
  min{Θ(G∘H),Θ(H∘G)}≥Θ(G)Θ(H).
  The next result, Proposition 3, gives a general graph–complement construction that is subsequently used in Proposition 4 to establish strict inequalities for several strongly regular graphs. Three countably infinite families of graph–complement pairs are subsequently obtained in Theorem 7 by taking lexicographic powers of these graphs; the strict inequality holds throughout these families, and the multiplicative gap is unbounded within each one.

**Proposition** **3.** *Let G be a finite simple graph on n vertices. Then,*

(85) $$\begin{array}{cc} \alpha \left({\left(G\circ \bar{G}\right)}^{⊠\;2}\right) & \geq n\;\alpha (G)\;\omega (G), \end{array}$$

(86) $$\begin{array}{cc} \alpha \left({\left(\bar{G}\circ G\right)}^{⊠\;2}\right) & \geq n\;\alpha (G)\;\omega (G). \end{array}$$

*Consequently,*

(87) $$\begin{array}{c} \min\left\{\Theta \left(G\circ \bar{G}\right),\;\Theta \left(\bar{G}\circ G\right)\right\}\geq \sqrt{n\;\alpha (G)\;\omega (G)}. \end{array}$$

*In particular, if*

(88) $$\begin{array}{c} \sqrt{n\;\alpha (G)\;\omega (G)}>\Theta \left(G\right)\;\Theta \left(\bar{G}\right), \end{array}$$

*then*

(89) $$\begin{array}{c} \min\left\{\Theta \left(G\circ \bar{G}\right),\;\Theta \left(\bar{G}\circ G\right)\right\}>\Theta \left(G\right)\;\Theta \left(\bar{G}\right). \end{array}$$

**Proof.**  Let *V* be the common vertex set of *G* and $\bar{G}$, with |V|=n. Choose maximum independent sets

$$A\subseteq V\left(G\right),\;B\subseteq V\left(\bar{G}\right).$$

Then, |A|=α(G) and $\left|B\right|=\alpha \left(\bar{G}\right)=\omega \left(G\right)$.

Consider the vertex subset of ${\left(G\circ \bar{G}\right)}^{⊠\;2}$ given by
  (90) $$\begin{array}{c} I≔\left\{\left((a,v),(v,b)\right):a\in A,\;v\in V,\;b\in B\right\}, \end{array}$$
  whose cardinality is
  |I|=nα(G)ω(G).
  We claim that I is an independent set in ${\left(G\circ \bar{G}\right)}^{⊠\;2}$. Let
  $$x=\left((a,v),(v,b)\right),\;x^{\prime }=\left(\left(a^{\prime },v^{\prime }\right),\left(v^{\prime },b^{\prime }\right)\right)$$
  be distinct elements of I. If $a\neq a^{\prime }$, then $a{≁}_{G}a^{\prime }$, since *A* is an independent set in *G*. Thus, (a,v) and $\left(a^{\prime },v^{\prime }\right)$ are distinct and nonadjacent in $G\circ \bar{G}$. If $a=a^{\prime }$ and $v\neq v^{\prime }$, then
  (91) $$\begin{array}{cc} \left(a,v\right){∼}_{G\circ \bar{G}}\left(a,v^{\prime }\right) & \;⟺\;v{∼}_{\bar{G}}v^{\prime }, \end{array}$$
  (92) $$\begin{array}{cc} \left(v,b\right){∼}_{G\circ \bar{G}}\left(v^{\prime },b^{\prime }\right) & \;⟺\;v{∼}_{G}v^{\prime }. \end{array}$$
  The two relations on the right cannot hold simultaneously. Finally, if $a=a^{\prime }$ and $v=v^{\prime }$, then $b\neq b^{\prime }$, and the independence of *B* in $\bar{G}$ implies that (v,b) and $\left(v,b^{\prime }\right)$ are distinct and nonadjacent in $G\circ \bar{G}$. Thus, in every case, *x* and $x^{\prime }$ are nonadjacent in the strong product. This proves (85).
For the reversed order, consider the vertex subset of ${\left(\bar{G}\circ G\right)}^{⊠2}$ given by
  (93) $$\begin{array}{c} J≔\left\{\left((b,v),(v,a)\right):b\in B,\;v\in V,\;a\in A\right\}, \end{array}$$
  whose cardinality is
  |J|=nα(G)ω(G).
  The same argument, with *G* and $\bar{G}$ interchanged, shows that J is an independent set in ${\left(\bar{G}\circ G\right)}^{⊠\;2}$. This proves (86).
By the definition of the Shannon capacity,
  $$\Theta \left(F\right)\geq \sqrt{\alpha (F^{⊠\;2})}$$
  for every finite graph *F*. Applying this inequality to $G\circ \bar{G}$ and $\bar{G}\circ G$ proves (87).
Combining (87) with condition (88) immediately yields (89). □

**Proposition** **4.** *Condition *(88)* holds for each of the following graphs and their complements:*

*1.*  *The Schläfli graph S=srg(27,16,10,8);*
*2.*  *The McLaughlin graph M=srg(275,112,30,56);*
*3.*  *The second subconstituent of the McLaughlin graph L=srg(162,56,10,24).*

**Proof.**  Condition (88) is verified for each of the three strongly regular graphs listed above.

1. For the Schläfli graph (see Section 10.10 of [38]),
  |V(S)|=27,α(S)=3,ω(S)=6,
  and
  $$\Theta \left(S\right)=3,\;\Theta \left(\bar{S}\right)\leq 7,$$
  where the first equality is given in Example 16 of [18] and the latter inequality follows from Haemers’ bound [15]. Hence,
  $$\begin{array}{cc} \Theta \left(S\right)\;\Theta \left(\bar{S}\right) & \leq \;21\\ \; & <\sqrt{\left|V(S)|\;\alpha (S)\;\omega (S\right)}=\sqrt{27\cdot 3\cdot 6}=9\sqrt{6}\approx 22.045. \end{array}$$
2. The McLaughlin graph (see Section 10.61 of [38]) has
  |V(M)|=275,α(M)=22,ω(M)=5,
  and adjacency spectrum
  $$112^{\left(1\right)},\;2^{\left(252\right)},\;{\left(-28\right)}^{\left(22\right)}.$$
  Let $A_{M}$ denote the adjacency matrix of *M*. The matrix $I-\frac{1}{2}A_{M}$ fits *M* over R: its diagonal entries are all equal to 1, while, for every pair of distinct nonadjacent vertices u,v∈V(M),
  $${\left(I-\frac{1}{2}A_{M}\right)}_{u,v}=-\frac{1}{2}{\left(A_{M}\right)}_{u,v}=0.$$
  Moreover, multiplication of this matrix by the nonzero scalar −2 does not change the rank. Therefore, Haemers’ rank bound [15] gives
  $$\Theta \left(M\right)\leq {\mathrm{rank}}_{R}\left(A_{M}-2I\right).$$
  Since 2 is an eigenvalue of $A_{M}$ with multiplicity 252, the nullity of $A_{M}-2I$ is 252. Consequently,
  $$\Theta \left(M\right)\leq {\mathrm{rank}}_{R}\left(A_{M}-2I\right)=275-252=23.$$
  The complement $\bar{M}$ is 162-regular and has least adjacency eigenvalue −3. For strongly regular graphs, the ratio bound for the Lovász theta function is attained (see Proposition 1 of [18]), which yields
  $$\Theta \left(\bar{M}\right)\leq \vartheta \left(\bar{M}\right)=\frac{275\cdot 3}{162+3}=5.$$
  Since also $\alpha \left(\bar{M}\right)=\omega \left(M\right)=5$, the equality $\Theta (\bar{M})=5$ follows, and consequently
  $$\begin{array}{cc} \Theta \left(M\right)\;\Theta \left(\bar{M}\right)\; & \leq 23\cdot 5=115\\ \; & <\sqrt{\left|V(M)|\;\alpha (M)\;\omega (M\right)}=\sqrt{275\cdot 22\cdot 5}=55\sqrt{10}\approx 173.925. \end{array}$$
3. Finally, let *L* be the second subconstituent of the McLaughlin graph (see Section 10.48 of [38]). It satisfies
  |V(L)|=162,α(L)=21,ω(L)=3,
  and has adjacency spectrum
  $$56^{\left(1\right)},\;2^{\left(140\right)},\;{\left(-16\right)}^{\left(21\right)}.$$
  Let $A_{L}$ denote its adjacency matrix. The matrix $A_{L}-2I$ fits *L* over R: its diagonal entries are all equal to −2 and hence are nonzero, whereas its entries corresponding to distinct nonadjacent vertices of *L* are zero. Therefore, Haemers’ rank bound [15] gives
  $$\Theta \left(L\right)\leq {\mathrm{rank}}_{R}\left(A_{L}-2I\right).$$
  Since 2 is an eigenvalue of $A_{L}$ with multiplicity 140, the nullity of $A_{L}-2I$ is 140. Consequently,
  $$\Theta \left(L\right)\leq {\mathrm{rank}}_{R}\left(A_{L}-2I\right)=162-140=22.$$
  The complement $\bar{L}$ is 105-regular and has least adjacency eigenvalue −3. For strongly regular graphs, the ratio bound for the Lovász theta function is attained (see Proposition 1 of [18]), which yields
  $$\Theta \left(\bar{L}\right)\leq \vartheta \left(\bar{L}\right)=\frac{162\cdot 3}{105+3}=\frac{9}{2}.$$
  It follows that
  $$\begin{array}{cc} \Theta \left(L\right)\;\Theta \left(\bar{L}\right) & \leq 22\cdot \frac{9}{2}=99\\ \; & <\sqrt{\left|V(L)|\;\alpha (L)\;\omega (L\right)}=\sqrt{162\cdot 21\cdot 3}=27\sqrt{14}\approx 101.025. \end{array}$$

Condition (88) is invariant under complementation, since complementation interchanges α(G) and ω(G) and leaves $\Theta \left(G\right)\;\Theta \left(\bar{G}\right)$ unchanged. The complements of the three listed graphs therefore satisfy the condition as well. □

**Corollary** **4.** *Let G be the Schläfli graph, the McLaughlin graph, or the second subconstituent of the McLaughlin graph. Then,*

(94) $$\begin{array}{c} \min\left\{\Theta \left(G\circ \bar{G}\right),\Theta \left(\bar{G}\circ G\right)\right\}>\Theta \left(G\right)\;\Theta \left(\bar{G}\right). \end{array}$$

*Consequently,*

(95) $$\begin{array}{cc} \Theta (G⊠\bar{G}) & >\Theta \left(G\right)\;\Theta \left(\bar{G}\right), \end{array}$$

(96) $$\begin{array}{cc} \Theta (G+\bar{G}) & >\Theta \left(G\right)+\Theta \left(\bar{G}\right). \end{array}$$

**Proof.**  The first assertion follows immediately from Propositions 3 and 4. More explicitly, the bounds obtained for the three graphs are

(97) $$\begin{array}{cc} \min\left\{\Theta \left(S\circ \bar{S}\right),\;\Theta \left(\bar{S}\circ S\right)\right\} & \geq 9\sqrt{6}>21\geq \Theta \left(S\right)\;\Theta \left(\bar{S}\right), \end{array}$$

(98) $$\begin{array}{cc} \min\left\{\Theta \left(M\circ \bar{M}\right),\;\Theta \left(\bar{M}\circ M\right)\right\} & \geq 55\sqrt{10}>115\geq \Theta \left(M\right)\;\Theta \left(\bar{M}\right), \end{array}$$

(99) $$\begin{array}{cc} \min\left\{\Theta \left(L\circ \bar{L}\right),\;\Theta \left(\bar{L}\circ L\right)\right\} & \geq 27\sqrt{14}>99\geq \Theta \left(L\right)\;\Theta \left(\bar{L}\right). \end{array}$$

For every pair of finite simple graphs *F* and *H*, the strong product F⊠H is a spanning subgraph of the lexicographic product F∘H; see Lemma 1. Since Shannon capacity is nonincreasing under the addition of edges,

Θ(F∘H)≤Θ(F⊠H).

Applying this inequality with F=G and $H=\bar{G}$, and using (94), gives (95).

Finally, the duality theorem established in [28,39] states that, for all finite simple graphs *F* and *H*,

(100) Θ(F+H)=Θ(F)+Θ(H)⟺Θ(F⊠H)=Θ(F)Θ(H).

Taking F=G and $H=\bar{G}$, inequality (95) implies that

$$\Theta \left(G+\bar{G}\right)\neq \Theta \left(G\right)+\Theta \left(\bar{G}\right).$$

Since the Shannon capacity is superadditive with respect to disjoint unions, the last inequality proves (96). □

**Remark** **3.** *The Schläfli graph recovers Alon’s example [40], whereas the McLaughlin graph and its second subconstituent provide two additional explicit graph–complement pairs for which Shannon capacity is strictly superadditive under disjoint union. Alon’s example disproved Shannon’s conjecture [1] that the Shannon capacity of a disjoint union is always equal to the sum of the capacities of its components.*

Building on Propositions 3 and 4, we construct three countably infinite families of graphs satisfying

(101) $$\begin{array}{c} \min\left\{\Theta \left(G\circ \bar{G}\right),\;\Theta \left(\bar{G}\circ G\right)\right\}>\Theta \left(G\right)\;\Theta \left(\bar{G}\right). \end{array}$$

Moreover, each family exhibits an arbitrarily large multiplicative gap: for every C>1, it contains a graph *G* for which the ratio of the left-hand side of (101) to its right-hand side is at least *C*.

**Theorem** **7.** *Let S, M, and L denote, respectively, the Schläfli graph, the McLaughlin graph, and the second subconstituent of the McLaughlin graph. For X∈{S,M,L} and an integer m≥1, let*

(102) $$\begin{array}{c} X_{m}≔X^{\circ m} \end{array}$$

*be the m-fold lexicographic power of X. Define*

(103) $$\begin{array}{cc} \; & G_{X}≔\left\{X_{m}:m\geq 1\right\},\;G≔G_{S}\cup G_{M}\cup G_{L}, \end{array}$$

(104) $$\begin{array}{cc} \; & R_{X}\left(m\right)≔\frac{\min\left\{\Theta \left(X_{m}\circ \bar{X_{m}}\right),\;\Theta \left(\bar{X_{m}}\circ X_{m}\right)\right\}}{\Theta \left(X_{m}\right)\;\Theta \left(\bar{X_{m}}\right)}. \end{array}$$

*Then, for all integers m≥1,*

(105) $$\begin{array}{cc} R_{S}\left(m\right) & \geq {\left(\frac{54}{49}\right)}^{m/2}, \end{array}$$

(106) $$\begin{array}{cc} R_{M}\left(m\right) & \geq {\left(\frac{1210}{529}\right)}^{m/2}, \end{array}$$

(107) $$\begin{array}{cc} R_{L}\left(m\right) & \geq {\left(\frac{126}{121}\right)}^{m/2}. \end{array}$$

*The right-hand side of each of these three inequalities is strictly larger than 1 for every m≥1 and tends to infinity as m→∞. Consequently, every graph in G satisfies *(101)*, and each of the three countably infinite families $G_{S}$, $G_{M}$, and $G_{L}$ exhibits an arbitrarily large multiplicative gap.*

**Proof.**  Fix X∈{S,M,L} and an integer m≥1. By the multiplicative formulas for the order, independence number, and clique number under lexicographic products (see Proposition 1),

(108) $$\left|V\left(X_{m}\right)\right|={\left|V\left(X\right)\right|}^{m},\;\alpha \left(X_{m}\right)=\alpha {\left(X\right)}^{m},\;\omega \left(X_{m}\right)=\omega {\left(X\right)}^{m}.$$

Applying Proposition 3 to $X_{m}$ gives

(109) $$\begin{array}{cc} \min\left\{\Theta \left(X_{m}\circ \bar{X_{m}}\right),\;\Theta \left(\bar{X_{m}}\circ X_{m}\right)\right\} & \geq \sqrt{\left|V\left(X_{m}\right)\right|\;\alpha \left(X_{m}\right)\;\omega \left(X_{m}\right)}\\ \; & ={\left(|V(X)|\;\alpha (X)\;\omega (X)\right)}^{m/2}. \end{array}$$

Furthermore, complementation commutes with the lexicographic product (see (13)), so $\bar{X_{m}}=\bar{X^{\circ m}}={\left(\bar{X}\right)}^{\circ m}.$ Hence, by Corollary 3,

$$\Theta \left(X_{m}\right)=\Theta {\left(X\right)}^{m},\;\Theta \left(\bar{X_{m}}\right)=\Theta {\left(\bar{X}\right)}^{m}.$$

Combining these identities with (103) and (109) yields

(110) $$\begin{array}{c} R_{X}\left(m\right)\geq {\left(\frac{\sqrt{\left|V(X)|\;\alpha (X)\;\omega (X\right)}}{\Theta \left(X\right)\;\Theta \left(\bar{X}\right)}\right)}^{m}. \end{array}$$

Using the graph parameters and the upper bounds on the relevant Shannon capacities established in the proof of Proposition 4, we obtain, for all integers m≥1,

$$\begin{array}{cc} R_{S}\left(m\right) & \geq {\left(\frac{\sqrt{27\cdot 3\cdot 6}}{3\cdot 7}\right)}^{m}={\left(\frac{54}{49}\right)}^{m/2},\\ R_{M}\left(m\right) & \geq {\left(\frac{\sqrt{275\cdot 22\cdot 5}}{23\cdot 5}\right)}^{m}={\left(\frac{1210}{529}\right)}^{m/2},\\ R_{L}\left(m\right) & \geq {\left(\frac{\sqrt{162\cdot 21\cdot 3}}{22\cdot \frac{9}{2}}\right)}^{m}={\left(\frac{126}{121}\right)}^{m/2}. \end{array}$$

These are precisely (105)–(107). Since $\frac{54}{49}>1,\;\frac{1210}{529}>1$, and $\frac{126}{121}>1$, the right-hand side of each inequality in (105)–(107) is strictly larger than 1 for every integer m≥1 and tends to infinity as m→∞. Therefore, every graph G∈G satisfies (101). More precisely, define

$$\rho_{S}≔\frac{54}{49}\approx 1.10204,\;\rho_{M}≔\frac{1210}{529}\approx 2.28733,\;\rho_{L}≔\frac{126}{121}\approx 1.04132.$$

Given C>1 and X∈{S,M,L}, choosing

$$m=\left\lceil \frac{2\ln C}{\ln\rho_{X}}\right\rceil$$

ensures that $R_{X}\left(m\right)\geq C$. Thus, for each of the families $G_{S}$, $G_{M}$, and $G_{L}$, the strict supermultiplicativity inequality (101) holds with an arbitrarily large multiplicative gap. Finally, each family is countably infinite. Indeed, for every X∈{S,M,L}, the family of graphs $G_{X}$ is indexed by the integers m≥1, and $\left|V\left(X_{m}\right)\right|={\left|V\left(X\right)\right|}^{m}$, which implies that $X_{m}$ and $X_{m^{\prime }}$ have different orders, and hence are nonisomorphic, whenever $m\neq m^{\prime }$. □

We now return to Theorem 6 and present some applications that yield exact evaluations of the Shannon capacity of lexicographic products. The following result determines this capacity for lexicographic products of Kneser-type graphs (see Section 2.5).

**Theorem** **8** (Lexicographic products of Kneser-type graphs)**.** *The following statements hold.*

*1.*  *If n≥2k and m≥2ℓ, then*
(111) ΘKG(n,k)∘KG(m,ℓ)=n−1k−1m−1ℓ−1.
*2.*  *If n≥2k, m≥2ℓ, k∣n, and ℓ∣m, then*
(112) $$\begin{array}{c} \Theta \left(\;\bar{\mathrm{KG}(n,k)}\circ \bar{\mathrm{KG}(m,\ell )}\;\right)=\frac{nm}{k\ell }. \end{array}$$
*3.*  *If q is a prime power, n≥2k, and m≥2ℓ, then*
(113) $$\Theta \left({\mathrm{KG}}_{q}\left(n,k\right)\circ {\mathrm{KG}}_{q}\left(m,\ell \right)\right)={\left[\;\begin{array}{c} \;n-1\\ \;k-1\; \end{array}\right]}_{q}{\left[\;\begin{array}{c} \;m-1\\ \;\ell -1\; \end{array}\right]}_{q}.$$

**Proof.**  We prove the three statements separately. In each case, the Shannon capacity and the Lovász theta number coincide for both factors. Theorem 6 then determines the Shannon capacity of their lexicographic product.

1. For classical Kneser graphs, Theorem 13 of [6] gives
  (114) ΘKG(n,k)=ϑKG(n,k)=n−1k−1,n≥2k,
  and, similarly,
  (115) ΘKG(m,ℓ)=ϑKG(m,ℓ)=m−1ℓ−1,m≥2ℓ.
  Therefore, Theorem 6 yields (111).
2. For complements of Kneser graphs, Theorem 2.51 of [5] gives
  (116) $$\begin{array}{c} \alpha \left(\bar{\mathrm{KG}(n,k)}\right)=\left\lfloor \frac{n}{k}\right\rfloor ,\;\vartheta \left(\bar{\mathrm{KG}(n,k)}\right)=\frac{n}{k}. \end{array}$$
  Thus, if k∣n, then
  (117) $$\begin{array}{c} \alpha \left(\bar{\mathrm{KG}(n,k)}\right)=\vartheta \left(\bar{\mathrm{KG}(n,k)}\right)=\frac{n}{k}. \end{array}$$
  Since α(F)≤Θ(F)≤ϑ(F) for every finite simple graph *F*, it follows that
  (118) $$\begin{array}{c} \Theta \left(\bar{\mathrm{KG}(n,k)}\right)=\vartheta \left(\bar{\mathrm{KG}(n,k)}\right)=\frac{n}{k}. \end{array}$$
  Analogously, if ℓ∣m, then
  (119) $$\begin{array}{c} \Theta \left(\bar{\mathrm{KG}(m,\ell )}\right)=\vartheta \left(\bar{\mathrm{KG}(m,\ell )}\right)=\frac{m}{\ell }. \end{array}$$
  Applying Theorem 6 to these two graphs gives (112).
3. For *q*-Kneser graphs, Theorem 6.2 of [5] gives
  (120) $$\begin{array}{c} \Theta \left({\mathrm{KG}}_{q}\left(n,k\right)\right)=\vartheta \left({\mathrm{KG}}_{q}\left(n,k\right)\right)={\left[\;\begin{array}{c} \;n-1\\ \;k-1\; \end{array}\right]}_{q}, \end{array}$$
  and, similarly, for ${\mathrm{KG}}_{q}\left(m,\ell \right)$. A final application of Theorem 6 therefore gives (113).

□

**Theorem** **9** (Repeated joins)**.** *For every finite simple graph G and every integer m≥1,*

(121) $$\begin{array}{c} \Theta \left(K_{m}\circ G\right)=\Theta \left(G\right). \end{array}$$

*Equivalently, for every integer m≥1,*

(122) $$\begin{array}{c} \Theta \left(\underset{m\;\mathrm{copies}}{\underset{︸}{G\vee G\vee \cdots \vee G}}\right)=\Theta \left(G\right). \end{array}$$

**Proof.**  Let the vertices of $K_{m}\circ G$ be written as pairs (i,v)∈[m]×V(G). Two such vertices (i,v) and (j,w) are adjacent if and only if i≠j or $\left(i=j\;\mathrm{and}\;v{∼}_{G}w\right)$. For n≥1, define the coordinate-wise projection

(123) $$\begin{array}{c} \pi :V\left({\left(K_{m}\circ G\right)}^{⊠n}\right)⟶V\left(G^{⊠n}\right) \end{array}$$

by

(124) $$\begin{array}{c} \pi \left(\left(i_{1},v_{1}\right),\ldots ,\left(i_{n},v_{n}\right)\right)=\left(v_{1},\ldots ,v_{n}\right). \end{array}$$

Let S be an independent set in ${\left(K_{m}\circ G\right)}^{⊠n}$.

First, π is injective on S. Indeed, suppose that two distinct words in S have the same image. In each coordinate their vertices are either equal, when their first coordinates agree, or adjacent, when their first coordinates differ. The two words would therefore be adjacent in the strong power, contradicting the independence of S.

Second, π(S) is independent in $G^{⊠n}$. Given two distinct words in S, their nonadjacency in the strong power gives a coordinate in which the corresponding vertices of $K_{m}\circ G$ are distinct and nonadjacent. Nonadjacency in $K_{m}\circ G$ forces their first coordinates to be equal and their *G*-coordinates to be nonadjacent. Hence, their projected words are nonadjacent in $G^{⊠n}$, and therefore

(125) $$\begin{array}{c} \alpha \left({\left(K_{m}\circ G\right)}^{⊠n}\right)\leq \alpha \left(G^{⊠n}\right). \end{array}$$

Conversely, choose one of the *m* copies of *G* in $K_{m}\circ G$, and in every factor of the strong product ${\left(K_{m}\circ G\right)}^{⊠n}$, allow only vertices belonging to that fixed copy. The resulting induced subgraph is isomorphic to $G^{⊠n}$. Therefore,

(126) $$\begin{array}{c} \alpha \left(G^{⊠n}\right)\leq \alpha \left({\left(K_{m}\circ G\right)}^{⊠n}\right), \end{array}$$

which proves the reverse inequality. Consequently, by (125) and (126), we obtain that, for all integers n≥1,

(127) $$\begin{array}{c} \alpha \left({\left(K_{m}\circ G\right)}^{⊠n}\right)=\alpha \left(G^{⊠n}\right). \end{array}$$

Taking the *n*-th roots of both sides of (127) and letting n→∞ proves (121). □

The next result provides another application of Theorem 6.

**Theorem** **10.** *Let G be a finite simple graph on n vertices.*

*1.*  *If G is either vertex-transitive or strongly regular, then*
(128) $$\begin{array}{c} \Theta (G\circ \bar{G})\leq n. \end{array}$$
*Moreover, equality holds if G is also self-complementary.*
*2.*  *If G is self-complementary, then for every integer m≥1,*
(129) $$\begin{array}{c} \Theta \left(G^{\;\circ \;m}\right)\geq n^{\frac{m}{2}}. \end{array}$$
*3.*  *If G is self-complementary and either vertex-transitive or strongly regular, then for every integer m≥1,*
(130) $$\begin{array}{c} \Theta \left(G^{\;\circ \;m}\right)=n^{\frac{m}{2}}. \end{array}$$

**Proof.**  Let *G* be a finite simple graph on *n* vertices.

1. Combining Theorem 6 of this paper and Theorem 3.26 (Item 1) of [10] shows that, if *G* is vertex-transitive or strongly regular, then
  (131) $$\begin{array}{c} \Theta \left(G\circ \bar{G}\right)\leq \Theta \left(G⊠\bar{G}\right)=n. \end{array}$$
  The equality assertion for self-complementary graphs will follow from the argument in Item 2.
2. If *G* is a self-complementary graph on *n* vertices, then
  (132) $$\begin{array}{cc} \Theta (G) & \geq \sqrt{\alpha (G⊠G)}\\ \; & =\sqrt{\alpha (G⊠\bar{G})}\\ \; & \geq \sqrt{n}, \end{array}$$
  where the first inequality holds by definition, the equality holds since $G≅\bar{G}$, and the last inequality holds since the diagonal set {(1,1),(2,2),…,(n,n)} is an independent set in $G⊠\bar{G}$. Hence, for every positive integer *m*,
  (133) $$\begin{array}{c} \Theta \left(G^{\;\circ \;m}\right)=\Theta {\left(G\right)}^{m}\geq n^{\frac{m}{2}}. \end{array}$$
  If *G* is self-complementary and either vertex-transitive or strongly regular on *n* vertices, then
  (134) $$\begin{array}{c} \Theta \left(G\circ \bar{G}\right)=\Theta \left(G\circ G\right)=\Theta {\left(G\right)}^{2}\geq n, \end{array}$$
  which, by combining with item 1, gives the equality
  (135) $$\begin{array}{c} \Theta (G\circ \bar{G})=n. \end{array}$$
  This proves the equality assertion in Item 1.
3. If the graph *G* is either self-complementary vertex-transitive or self-complementary strongly regular, then by Item 1,
  (136) $$\begin{array}{c} \Theta {\left(G\right)}^{2}=\Theta \left(G\circ G\right)=\Theta \left(G\circ \bar{G}\right)\leq n, \end{array}$$
  so $\Theta \left(G\right)\leq \sqrt{n}$. Combining this inequality with Item 2 gives that $\Theta \left(G\right)=\sqrt{n}$. Consequently, for every integer m≥1,
  (137) $$\begin{array}{c} \Theta \left(G^{\;\circ \;m}\right)=\Theta {\left(G\right)}^{m}=n^{\frac{m}{2}}. \end{array}$$

□

**Example** **4.** *Let P(n) be a Paley graph on n vertices, where n is a prime power satisfying n≡1(mod4). This graph is self-complementary, vertex-transitive, and strongly regular (see Section 2.6). Consequently, by Item 3 of Theorem 10,*

(138) $$\begin{array}{c} \Theta \left(P{\left(n\right)}^{\;\circ \;m}\right)=n^{\frac{m}{2}}. \end{array}$$

*In particular, we have*

(139) $$\begin{array}{c} \Theta \left(C_{5}^{\;\circ \;m}\right)=5^{\frac{m}{2}}, \end{array}$$

*where $C_{5}≅P\left(5\right)$ denotes the pentagon (see Example 3).*

Combining Theorems 2 and 6, together with the multiplicativity property of the fractional Haemers number under strong products (see Theorem 3 of [16]), the following bounds on the Shannon capacity of a lexicographic product of graphs are readily obtained.

**Theorem** **11** (Bounds for the Shannon capacity)**.** *For all finite simple graphs G and H, and for every finite field F,*

(140) $$\begin{array}{cc} \Theta (G)\;\Theta (H) & \leq \min\{\Theta (G\circ H),\Theta (H\circ G)\}\\ \; & \leq \max\{\Theta (G\circ H),\Theta (H\circ G)\}\\ \; & \leq \Theta (G⊠H)\\ \; & \leq H_{f}\left(G;F\right)\;H_{f}\left(H;F\right). \end{array}$$

**Example** **5.** *Let G be the Schläfli graph, and let n≥1 be an integer. By Corollary 3, since α(G)=Θ(G)=ϑ(G)=3, we have*

$$\Theta \left(G^{\;\circ n}\right)=\Theta {\left(G\right)}^{n}=3^{n}.$$

*Thus, the upper bound in Theorem 6 is tight in this case. By contrast, Theorem 11 gives*

$$\Theta \left(G^{\;\circ n}\right)\leq H_{f}{\left(G;R\right)}^{n}\leq \chi_{f}{\left(\bar{G}\right)}^{n}={\left(\frac{9}{2}\right)}^{n}.$$

*Here, we have used the general inequality*

(141) $$\begin{array}{c} H_{f}\left(F;F\right)\leq \chi_{f}\left(\bar{F}\right), \end{array}$$

*which holds for every finite simple graph F and every field F, where $\chi_{f}\left(\cdot \right)$ denotes the fractional chromatic number of the graph. Moreover, since $\bar{G}$ is vertex-transitive and $\alpha \left(\bar{G}\right)=\omega \left(G\right)=6$,*

(142) $$\begin{array}{c} \chi_{f}\left(\bar{G}\right)=\frac{\left|V(G)\right|}{\alpha (\bar{G})}=\frac{27}{6}=\frac{9}{2}. \end{array}$$

*For the complement of the Schläfli graph $\bar{G}$, the situation is reversed. Theorem 6 gives*

(143) $$\begin{array}{c} \Theta \left({\bar{G}}^{\;\circ n}\right)\leq \vartheta {\left(\bar{G}\right)}^{n}=9^{n}, \end{array}$$

*whereas Theorem 11 gives, for every field F,*

(144) $$\begin{array}{c} \Theta \left({\bar{G}}^{\;\circ n}\right)\leq H_{f}{\left(\bar{G};F\right)}^{n}\leq 7^{n}. \end{array}$$

*Hence, for $\bar{G}$, Theorem 11 provides a strictly sharper upper bound on the Shannon capacity of its n-fold lexicographic power. This shows that the upper bounds in Theorems 6 and 11 are incomparable in general.*

## 6. An Open Problem on Graph-Product Capacities

Theorem 6 shows that the Shannon capacity of each of the two ordered lexicographic products is at most that of the strong product. Motivated by the strict supermultiplicativity constructions in Theorem 7, we ask whether there exist finite simple graphs *G* and *H* such that

(145) $$\max\left\{\Theta (G\circ H),\;\Theta (H\circ G)\right\}<\Theta \left(G⊠H\right).$$

Equivalently, can the Shannon capacity of the strong product of two finite simple graphs be strictly larger than the capacities of both corresponding ordered lexicographic products?

While this question remains open, we discuss a possible approach to resolving it based on the results of this paper.

Propositions 3 and 4 suggest several natural candidates. Let *S*, *M*, and *L* denote, respectively, the Schläfli graph, the McLaughlin graph, and the second subconstituent of the McLaughlin graph. These are vertex-transitive strongly regular graphs with parameters

(146) $$\begin{array}{cc} S & =\mathrm{srg}(27,16,10,8), \end{array}$$

(147) $$\begin{array}{cc} M & =\mathrm{srg}(275,112,30,56), \end{array}$$

(148) $$\begin{array}{cc} L & =\mathrm{srg}(162,56,10,24). \end{array}$$

If *X* is a vertex-transitive or strongly regular graph on *n* vertices, then Theorem 3.26 of [10] gives

(149) $$\begin{array}{c} \alpha \left(X⊠\bar{X}\right)=\Theta \left(X⊠\bar{X}\right)=\vartheta \left(X⊠\bar{X}\right)=n. \end{array}$$

In particular, (149) holds for every X∈{S,M,L}. For each of these graphs, define

(150) $$\begin{array}{c} b_{X}≔\sqrt{\left|V(X)|\;\alpha (X)\;\omega (X\right)}. \end{array}$$

The relevant parameters and bounds are summarized in Table 1. Here, $U_{X}$ denotes the upper bound on $\Theta \left(X\right)\;\Theta \left(\bar{X}\right)$ established in Proposition 4.

Thus, for every X∈{S,M,L},

(151) $$\begin{array}{c} \Theta \left(X\right)\;\Theta \left(\bar{X}\right)\leq U_{X}<b_{X}. \end{array}$$

On the other hand, Proposition 3 and Theorem 6 give

(152) $$\begin{array}{cc} b_{X} & \leq \min\left\{\Theta \left(X\circ \bar{X}\right),\;\Theta \left(\bar{X}\circ X\right)\right\}\\ \; & \leq \max\left\{\Theta \left(X\circ \bar{X}\right),\;\Theta \left(\bar{X}\circ X\right)\right\}\\ \; & \leq \Theta \left(X⊠\bar{X}\right)=\left|V\left(X\right)\right|. \end{array}$$

Consequently, the available bounds are

(153) $$\begin{array}{cc} 9\sqrt{6} & \leq \min\left\{\Theta \left(S\circ \bar{S}\right),\;\Theta \left(\bar{S}\circ S\right)\right\}\\ \; & \leq \max\left\{\Theta \left(S\circ \bar{S}\right),\;\Theta \left(\bar{S}\circ S\right)\right\}\leq 27, \end{array}$$

(154) $$\begin{array}{cc} 55\sqrt{10} & \leq \min\left\{\Theta \left(M\circ \bar{M}\right),\;\Theta \left(\bar{M}\circ M\right)\right\}\\ \; & \leq \max\left\{\Theta \left(M\circ \bar{M}\right),\;\Theta \left(\bar{M}\circ M\right)\right\}\leq 275, \end{array}$$

(155) $$\begin{array}{cc} 27\sqrt{14} & \leq \min\left\{\Theta \left(L\circ \bar{L}\right),\;\Theta \left(\bar{L}\circ L\right)\right\}\\ \; & \leq \max\left\{\Theta \left(L\circ \bar{L}\right),\;\Theta \left(\bar{L}\circ L\right)\right\}\leq 162. \end{array}$$

It is currently unknown whether, for any X∈{S,M,L},

$$\max\left\{\Theta \left(X\circ \bar{X}\right),\;\Theta \left(\bar{X}\circ X\right)\right\}<\left|V\left(X\right)\right|.$$

A strict inequality in any one of these three cases would resolve the open problem posed in (145).

It is also worth comparing these bounds with the one-shot independence numbers. By the multiplicativity of the independence number under lexicographic products,

(156) $$\alpha \left(X\circ \bar{X}\right)=\alpha \left(\bar{X}\circ X\right)=\alpha \left(X\right)\;\alpha \left(\bar{X}\right)=\alpha \left(X\right)\;\omega \left(X\right).$$

Since α(X)ω(X)<|V(X)| for each X∈{S,M,L}, we have

$$\alpha \left(X\right)\;\omega \left(X\right)<\sqrt{\left|V(X)|\;\alpha (X)\;\omega (X\right)}=b_{X},$$

and therefore

(157) $$\begin{array}{c} \alpha \left(X\circ \bar{X}\right)=\alpha \left(\bar{X}\circ X\right)<b_{X}\leq \min\left\{\Theta \left(X\circ \bar{X}\right),\;\Theta \left(\bar{X}\circ X\right)\right\}. \end{array}$$

Thus, for each of the three candidate graphs, the Shannon capacities of $X\circ \bar{X}$ and $\bar{X}\circ X$ are both strictly larger than their common independence number. By contrast, (149) shows that the Shannon capacity of $X⊠\bar{X}$ is attained by its independence number.

More generally, Theorem 7 provides three countably infinite families of candidate graph–complement pairs. For X∈{S,M,L} and an integer m≥1, let $X_{m}≔X^{\;\circ m}$, as in Theorem 7. Since lexicographic powers of vertex-transitive graphs are vertex-transitive, it follows that $X_{m}$ is vertex-transitive, and

(158) $$\begin{array}{c} \Theta \left(X_{m}⊠\bar{X_{m}}\right)=\left|V\left(X_{m}\right)\right|={\left|V\left(X\right)\right|}^{m}. \end{array}$$

At the same time, Theorem 7 gives

(159) $$\begin{array}{c} \min\left\{\Theta \left(X_{m}\circ \bar{X_{m}}\right),\;\Theta \left(\bar{X_{m}}\circ X_{m}\right)\right\}>\Theta \left(X_{m}\right)\;\Theta \left(\bar{X_{m}}\right). \end{array}$$

It remains open whether, for some graph X∈{S,M,L} and some integer m≥1,

(160) $$\begin{array}{cc} \max\left\{\Theta \left(X_{m}\circ \bar{X_{m}}\right),\;\Theta \left(\bar{X_{m}}\circ X_{m}\right)\right\}\; & <\Theta (X_{m}⊠\bar{X_{m}}). \end{array}$$

A positive answer would resolve the open problem posed in (145).

## Figures and Tables

**Table 1:** Parameters and capacity bounds for the candidate graphs.

*X*	|V(X)|	α(X)	ω(X)	$b_{X}$	$U_{X}$
*S*	27	3	6	$9\sqrt{6}\approx 22.045$	21
*M*	275	22	5	$55\sqrt{10}\approx 173.925$	115
*L*	162	21	3	$27\sqrt{14}\approx 101.025$	99

## Data Availability

The original contributions presented in this study are included in the article. Further inquiries can be directed to the corresponding author.
